# Trophectoderm differentiation to invasive syncytiotrophoblast is promoted by endometrial epithelial cells during human embryo implantation

**DOI:** 10.1093/humrep/deac008

**Published:** 2022-01-26

**Authors:** Peter T Ruane, Terence Garner, Lydia Parsons, Phoebe A Babbington, Ivan Wangsaputra, Susan J Kimber, Adam Stevens, Melissa Westwood, Daniel R Brison, John D Aplin

**Affiliations:** 1 Faculty of Biology, Medicine and Health, Division of Developmental Biology and Medicine, Maternal and Fetal Health Research Centre, School of Medical Sciences, Saint Mary’s Hospital, Manchester Academic Health Sciences Centre, University of Manchester, Manchester, UK; 2 Maternal and Fetal Health Research Centre, Saint Mary’s Hospital, Manchester University NHS Foundation Trust, Manchester Academic Health Sciences Centre, Manchester, UK; 3 Department of Reproductive Medicine, Old Saint Mary’s Hospital, Manchester University NHS Foundation Trust, Manchester Academic Health Science Centre, Manchester, UK; 4 Faculty of Biology Medicine and Health, Division of Cell Matrix Biology and Regenerative Medicine, School of Biological Sciences, University of Manchester, Manchester, UK

**Keywords:** implantation, embryo development, trophoblasts, gene expression, cell culture

## Abstract

**STUDY QUESTION:**

How does the human embryo breach the endometrial epithelium at implantation?

**SUMMARY ANSWER:**

Embryo attachment to the endometrial epithelium promotes the formation of multinuclear syncytiotrophoblast from trophectoderm, which goes on to breach the epithelial layer.

**WHAT IS KNOWN ALREADY:**

A significant proportion of natural conceptions and assisted reproduction treatments fail due to unsuccessful implantation. The trophectoderm lineage of the embryo attaches to the endometrial epithelium before breaching this barrier to implant into the endometrium. Trophectoderm-derived syncytiotrophoblast has been observed in recent *in vitro* cultures of peri-implantation embryos, and historical histology has shown invasive syncytiotrophoblast in embryos that have invaded beyond the epithelium, but the cell type mediating invasion of the epithelial layer at implantation is unknown.

**STUDY DESIGN, SIZE, DURATION:**

Fresh and frozen human blastocyst-stage embryos (n = 46) or human trophoblast stem cell (TSC) spheroids were co-cultured with confluent monolayers of the Ishikawa endometrial epithelial cell line to model the epithelial phase of implantation *in vitro*. Systems biology approaches with published transcriptomic datasets were used to model the epithelial phase of implantation *in silico*.

**PARTICIPANTS/MATERIALS, SETTING, METHODS:**

Human embryos surplus to treatment requirements were consented for research. Day 6 blastocysts were co-cultured with Ishikawa cell layers until Day 8, and human TSC spheroids modelling blastocyst trophectoderm were co-cultured with Ishikawa cell layers for 48 h. Embryo and TSC morphology was assessed by immunofluorescence microscopy, and TSC differentiation by real-time quantitative PCR (RT-qPCR) and ELISA. Single-cell human blastocyst transcriptomes, and bulk transcriptomes of TSC and primary human endometrial epithelium were used to model the trophectoderm–epithelium interaction *in silico*. Hypernetworks, pathway analysis, random forest machine learning and RNA velocity were employed to identify gene networks associated with implantation.

**MAIN RESULTS AND THE ROLE OF CHANCE:**

The majority of embryos co-cultured with Ishikawa cell layers from Day 6 to 8 breached the epithelial layer (37/46), and syncytiotrophoblast was seen in all of these. Syncytiotrophoblast was observed at the embryo-epithelium interface before breaching, and syncytiotrophoblast mediated all pioneering breaching events observed (7/7 events). Multiple independent syncytiotrophoblast regions were seen in 26/46 embryos, suggesting derivation from different regions of trophectoderm. Human TSC spheroids co-cultured with Ishikawa layers also exhibited syncytiotrophoblast formation upon invasion into the epithelium. RT-qPCR comparison of TSC spheroids in isolated culture and co-culture demonstrated epithelium-induced upregulation of syncytiotrophoblast genes CGB (*P* = 0.03) and SDC1 (*P* = 0.008), and ELISA revealed the induction of hCGβ secretion (*P* = 0.03). Secretory-phase primary endometrial epithelium surface transcriptomes were used to identify trophectoderm surface binding partners to model the embryo-epithelium interface. Hypernetwork analysis established a group of 25 epithelium-interacting trophectoderm genes that were highly connected to the rest of the trophectoderm transcriptome, and epithelium-coupled gene networks in cells of the polar region of the trophectoderm exhibited greater connectivity (*P* < 0.001) and more organized connections (*P* < 0.0001) than those in the mural region. Pathway analysis revealed a striking similarity with syncytiotrophoblast differentiation, as 4/6 most highly activated pathways upon TSC-syncytiotrophoblast differentiation (false discovery rate (FDR < 0.026)) were represented in the most enriched pathways of epithelium-coupled gene networks in both polar and mural trophectoderm (FDR < 0.001). Random forest machine learning also showed that 80% of the endometrial epithelium-interacting trophectoderm genes identified in the hypernetwork could be quantified as classifiers of TSC-syncytiotrophoblast differentiation. This multi-model approach suggests that invasive syncytiotrophoblast formation from both polar and mural trophectoderm is promoted by attachment to the endometrial epithelium to enable embryonic invasion.

**LARGE SCALE DATA:**

No omics datasets were generated in this study, and those used from previously published studies are cited.

**LIMITATIONS, REASONS FOR CAUTION:**

*In vitro* and *in silico* models may not recapitulate the dynamic embryo-endometrial interactions that occur *in vivo*. The influence of other cellular compartments in the endometrium, including decidual stromal cells and leukocytes, was not represented in these models.

**WIDER IMPLICATIONS OF THE FINDINGS:**

Understanding the mechanism of human embryo breaching of the epithelium and the gene networks involved is crucial to improve implantation success rates after assisted reproduction. Moreover, early trophoblast lineages arising at the epithelial phase of implantation form the blueprint for the placenta and thus underpin foetal growth trajectories, pregnancy health and offspring health.

**STUDY FUNDING/COMPETING INTEREST(S):**

This work was funded by grants from Wellbeing of Women, Diabetes UK, the NIHR Local Comprehensive Research Network and Manchester Clinical Research Facility, and the Department of Health Scientist Practitioner Training Scheme. None of the authors has any conflict of interest to declare.

## Introduction

Pregnancy is established as the embryo implants into the endometrium, whereupon placentation enables foetal development. Trophectoderm (TE) forms the outer cell layer of the blastocyst-stage embryo, attaching to the receptive luminal epithelium of the endometrium to initiate implantation before TE-derived trophoblast penetrates the epithelium and invades the endometrial stroma (Aplin and Ruane, 2017). Recent progress with *in vitro* culture has revealed details of the cellular choreography of peri-implantation human embryos, up to Day 14, in isolation (Deglincerti *et al.*, 2016; Shahbazi *et al.*, 2016, 2017; Popovic *et al.*, 2019; West *et al.*, 2019; Zhou *et al.*, 2019; Xiang *et al.*, 2020), and the effects of maternal cell interactions on peri-implantation development up to Day 10 have been explored using endometrial stromal cells (Lv *et al.*, 2019). However, breaching of the endometrial epithelium in the first phase of human embryo implantation remains largely uncharacterized.

Human embryo implantation *in situ* has been observed only from the early stromal invasion phase in historic samples (Hertig *et al.*, 1956). Many studies have reported interaction of the human blastocyst with endometrial epithelial cells (EEC) *in vitro* (Lindenberg *et al.*, 1985; Bentin-Ley *et al.*, 2000; Galan *et al.*, 2000; Meseguer *et al.*, 2001; Petersen *et al.*, 2005; Lalitkumar *et al.*, 2007, 2013; Kang *et al.*, 2014; Berger *et al.*, 2015; Boggavarapu *et al.*, 2016; Aberkane *et al.*, 2018; Ruane *et al.*, 2020), however, these have predominantly focussed on blastocyst attachment with limited analysis of cellular morphology. Ultrastructural analysis of six blastocysts attached to EEC *in vitro* demonstrated shared desmosomal junctions mediating TE-EEC attachment, TE invasion between EEC and bi-nucleated TE cells potentially representing syncytiotrophoblast (STB) (Bentin-Ley *et al.*, 2000). Trophoblast develops from TE during implantation, and STB forms by cell fusion to produce multinucleated cells. STB was seen to mediate early stromal invasion at human implantation *in vivo* (Hertig *et al.*, 1956), and in earlier implantation samples in non-human primates it was observed to penetrate the endometrial epithelium (Enders *et al.*, 1983; Smith *et al.*, 1987). STB has been seen in human embryos developing beyond the blastocyst stage in isolation (Deglincerti *et al.*, 2016; Shahbazi *et al.*, 2016; West *et al.*, 2019) and in culture with endometrial cells (Aberkane *et al.*, 2018; Lv *et al.*, 2019), but whether it mediates embryo breaching of the endometrial epithelium in human remains unknown. Our previous study in mouse embryos suggested that interaction with EEC stimulates trophoblast differentiation (Ruane *et al.*, 2017), implicating the epithelial phase of implantation as important for trophoblast development.

The maternal-facing cells of the placenta derive from the TE, and as such a subpopulation of trophoblast must retain multipotency during implantation (Knofler *et al.*, 2019), while post-mitotic trophoblast invades the endometrium (Chuprin *et al.*, 2013; Lu *et al.*, 2017; Velicky *et al.*, 2018). Multipotent human trophoblast stem cells (TSCs) have recently been derived from blastocysts and first trimester placenta (Okae *et al.*, 2018), and from naïve embryonic stem cells (Dong *et al.*, 2020), providing promising systems to study implantation. In mice, the pioneering invasive trophoblast derives from the subset of the TE not in contact with the inner cell mass (ICM) of the blastocyst, termed mural TE, while the ICM-adjacent TE, termed polar TE, gives rise to multipotent trophoblast of the ectoplacental cone (Sutherland, 2003). *In vitro* studies have demonstrated that human blastocysts orient such that polar TE may mediate attachment to EEC or adhesive surfaces (Bentin-Ley *et al.*, 2000; Deglincerti *et al.*, 2016; Shahbazi *et al.*, 2016; Aberkane *et al.*, 2018), while it is not clear from specimens of later stages of human implantation *in vivo* whether polar TE initiates invasion into the endometrium (Hertig *et al.*, 1956). Differences between human polar and mural TE have been described at the transcriptomic level (Petropoulos *et al.*, 2016; Meistermann *et al.*, 2021), but little is understood about differential function of these TE subpopulations.

We and others have recently characterized human blastocyst attachment to the endometrial epithelial Ishikawa cell line. This revealed that both clinical embryology grading of TE morphology and hatching from the zona pellucida affect blastocyst attachment (Ruane *et al.*, 2020), while adhesion-related genes are upregulated in attached embryos (Aberkane *et al.*, 2018). Here, we further interrogate embryo-EEC interactions *in vitro* and show that STB mediates embryonic breaching of the epithelial layer. Moreover, our TSC spheroid and *in silico* models indicate that attachment to EEC promotes STB differentiation.

## Materials and methods

### Human embryos

Embryos generated for IVF treatment but not used by patients were obtained with informed written consent at Old Saint Marys Hospital, Manchester, or other IVF units in England. Embryos from both current and previous (cryopreserved) treatment cycles were used in accordance with ethics approval from the NRES committee south central (Berkshire; REC reference: 12/SC/0649), and a research licence from the Human Fertilisation and Embryology Authority (R0026; Person Responsible: Daniel Brison), centre 0067 (Old Saint Mary’s Hospital; fresh embryo research) and University of Manchester (0175; frozen-thawed embryo research). Day 6 blastocysts were artificially hatched using acid Tyrode (pH 2.5), as described (Ruane *et al.*, 2020), before use. Forty-six blastocysts (expanded morphology at Day 6 and of unknown clinical grade) were used in this study; four of the embryos were thawed from cryopreservation at pro-nuclear stage or on Day 2 after fertilization (clinical morphology grades were not assessed). The remaining 42 embryos were donated as blastocysts fresh from IVF cycles and were unsuitable for freezing, with grades below 2/5 for expansion, 4/5 for ICM and 2/3 for TE (Cutting *et al.*, 2008; ASiRMaESIGo, 2011).

### Ishikawa cell culture

Ishikawa EEC (ECACC 99040201) were cultured at 37°C, 95% air and 5% CO_2_ in growth medium (1:1 Dulbecco’s modified Eagle’s medium: Ham’s-F12 (Sigma) containing 10% v/v foetal bovine serum (Gibco) and supplemented with 2 mM L-glutamine, 100 µg/ml streptomycin and 100 IU/ml penicillin (Sigma)). Ishikawa cells were grown to confluency to form layers in 24-well plates (Greiner) on 13 mm glass coverslips for co-culture with blastocysts and TSC spheroids and subsequent microscopy, or in 48-well plates without coverslips for co-culture with TSC spheroids for subsequent gene expression and ELISA analysis.

### Human blastocyst implantation assay

Confluent Ishikawa EEC layers were washed and replenished with serum-free growth medium immediately prior to the transfer of single hatched Day 6 blastocysts to individual wells. After 48 h, all co-cultures were washed in PBS and fixed with 4% w/v paraformaldehyde (PFA) in PBS for 20 min.

### TSC culture and spheroid formation

TSC (line CT27, derived from first trimester placenta) were acquired as a gift from Dr Hiroake Okae, and cultured and differentiated to adherent STB as described (Okae *et al.*, 2018). TSC were seeded into Aggrewell™ plates (Stem Cell Technologies) at ∼200 cells/microwell and cultured in serum- and BSA-free TSC medium for 48 h to produce spheroids.

### TSC spheroid implantation assay

TSC spheroids were collected from Aggrewell™ plates and resuspended in co-culture medium (1:1 DMEM: Ham’s-F12, 1% ITS-X (Gibco), 5 µM Y27632 (Adooq Bioscience), 2 mM L-glutamine and 100 µg/ml streptomycin and 100 IU/ml penicillin). Approximately 20 spheroids, pre-treated with CellTracker™ (Life Technologies), were co-cultured per well of Ishikawa cells in 24-well plates for 48 h before fixation with 4% PFA. Attachment was monitored over the first 6 h of co-culture by agitating the culture plate to determine which spheroids were attached to the Ishikawa EEC layer. Alternatively, ∼400 spheroids per well of Ishikawa cells in 48-well plates were co-cultured for 48 h, before lysis and RNA extraction.

### Staining and fluorescence imaging

Fixed samples were quenched with 50 mM ammonium chloride solution before permeabilization with 0.5% Triton-X 100 PBS. Samples were incubated with primary antibody in PBS for 2 h or overnight at 4°C, followed by alexa568-phalloidin (Life Technologies), 4′,6-diamidino-2-phenylindole (DAPI) (Sigma) and alexa488/649 secondary antibodies (Life Technologies) for 1 h. Human embryo samples were mounted in a chamber of 3% 1,4-diazabicyclo[2.2.2]octane (DABCO) (Sigma) in PBS. TSC samples were mounted on a glass slide with 3% DABCO Mowiol (Sigma). Fluorescence microscopy was carried out using a Zeiss Axiophot microscope equipped with an Apotome module for optical sectioning. Images were processed using Zeiss Zen software and ImageJ. Antibodies: rabbit anti-E-cadherin (EP700Y, Abcam), rabbit anti-CDX2 (D11D10, Cell Signalling Technologies), mouse anti-GATA3 (MAB6330, R&D Systems), mouse anti-hCGβ (5H4-E2, Abcam) and mouse anti-HLA-G (4H84, Abcam).

### RNA extraction and PCR

RNA was extracted using the RNeasy Mini Kit (Qiagen), according to the manufacturer’s instructions. Reverse transcription using random 9mer primers (Agilent) followed protocols for Sensiscript Reverse Transcription kit (Qiagen). Real-time quantitative PCR (RT-qPCR) were performed using reverse transcription reactions together with 0.25 µM gene-specific primers (*SDC1* CTGCCGCAAATTGTGGCTAC, TGAGCCGGAGAAGTTGTCAGA; CGB CAGCATCCTATCACCTCCTGGT, CTGGAACATCTCCATCCTTGGT; OVOL1 TGAACCGCCACATGAAGTGTC, GACGTGTCTCTTGAGGTCGAA; ACTB AAGCCACCCCACTTCTCTCT, CTATCACCTCCCCTGTGTGG) and QuantiTect SYBR green PCR kit (Qiagen) on StepOne Plus machines; 40 cycles, 60°C annealing temperature. Raw data were analysed with StepOne Plus software to yield cycle threshold (Ct) values, which were expressed as 2^−ct^ relative to housekeeping gene, *ACTB*. In order to normalize total transcript levels of TSC spheroids cultured in isolation to that of transcripts from TSC spheroids cultured with Ishikawa cells, reverse transcribed RNA from samples of isolated culture TSC spheroids were pooled with reverse transcribed RNA extracts from samples of isolated Ishikawa cultures before analysis by RT-qPCR.

### ELISA

Media from TSC spheroid cultures and co-cultures were collected and subject to hCGβ ELISA using kit EIA-1496 (DRG Instruments GmbH), according to the manufacturer’s instructions.

### Modelling the TE-EEC interface in silico

All *in silico* modelling and analysis was performed in R version 3.4.2 (R Foundation for Statistical Computing). Differentially expressed genes (DEGs) were defined by ANOVA (cut-off *P* < 0.01) between proliferative phase (n = 4) and mid-secretory-phase (n = 4) endometrial epithelial RNA sequencing (RNAseq) transcriptomes (GEO accession number GSE132711) (Chi *et al.*, 2020). Localization of the protein products of these genes was investigated using the Database for Annotation, Visualization and Integrated Discovery (DAVID) (Huang da *et al.*, 2009a,b). Proteins defined as localized to any of ‘extracellular space’, ‘extracellular matrix’, ‘proteinaceous extracellular matrix’ or ‘cell surface’ were refined from the list of DEGs. Putative binding partners were identified using the Biological General Repository for Interaction Datasets (BioGRID) (Stark *et al.*, 2006) and refined to those which were localized as above in Day 6 TE single cell-RNAseq transcriptomes (n = 331) (Array Express Accession E-MTAB-3929) (Petropoulos *et al.*, 2016). Transcriptomic differences between samples were visualized by principal component analysis (PCA) and partial least-squares-discriminant analysis (PLS-DA). All pathway analyses were performed using Webgestalt (Zhang *et al.*, 2005), and word clouds were generated using WocEA (Ning *et al.*, 2018).

### Hypernetworks

Hypernetworks were used to quantify shared correlations between EEC-interacting TE surface genes across the rest of the transcriptome. Correlation matrices were generated using the Day 6 TE single cell-RNAseq transcriptomes. All analyses were repeated for polar and mural TE separately, modifying the samples included in each experiment, using previously inferred TE subpopulations (Petropoulos *et al.*, 2016). Correlation matrices were binarized using the distribution of *r*-values in order to include only the strongest present correlations. Correlations further from the mean *r*-value than ±1 SD in each set were assigned ‘1’, while those between the SD boundaries was assigned ‘0’. Hypernetworks were generated by multiplying the binary matrix (M) by the transpose of that matrix ($M^{t}$) to describe the number of shared correlations between a pair of TE genes. The central cluster of the hypernetworks, arranged using hierarchical clustering, represent a subset of EEC-interacting TE genes whose expression patterns are correlated with a large number of genes across the transcriptome. The connectivity (mean number of shared connections) and organization (Shannon entropy) of the central clusters were quantified. These metrics were also calculated by permuting 1000 hypernetworks of random genes within the transcriptome.

### Classification of genes using random forest

EEC-interacting TE genes present in TSC and TSC-derived STB (Okae *et al.*, 2018) were used for random forest (RF) machine learning (Breiman, 2001), which was applied over 1000 iterations to test whether the genes could accurately classify STB differentiation. Noise within the RF was modelled using Boruta (Kursa and Rudnicki, 2010).

### RNA velocity

RNA velocity analysis was performed on TE cells of Day 5 embryo cells, and mural TE and polar TE cells of Day 6 and 7 embryos (Petropoulos *et al.*, 2016), using the scVelo package (La Manno *et al.*, 2018; Bergen *et al.*, 2020). The inferred velocity of genes identified in the hypernetwork analysis were then compared between these TE cells, with velocity (*v*) plotted as $\frac{V}{\left|\mathrm{|V|}\right|}ln(1+\left|\mathrm{|V|}\right|)$.

### Statistical comparisons

Statistical significance was measured by Chi-squared and Mann–Whitney analyses using Prism (GraphPad). ANOVA and Wilcoxon rank sum analyses were performed using R.

## Results

### Gross and cellular morphology of embryos attached to Ishikawa EEC layers

**Figure 1.**
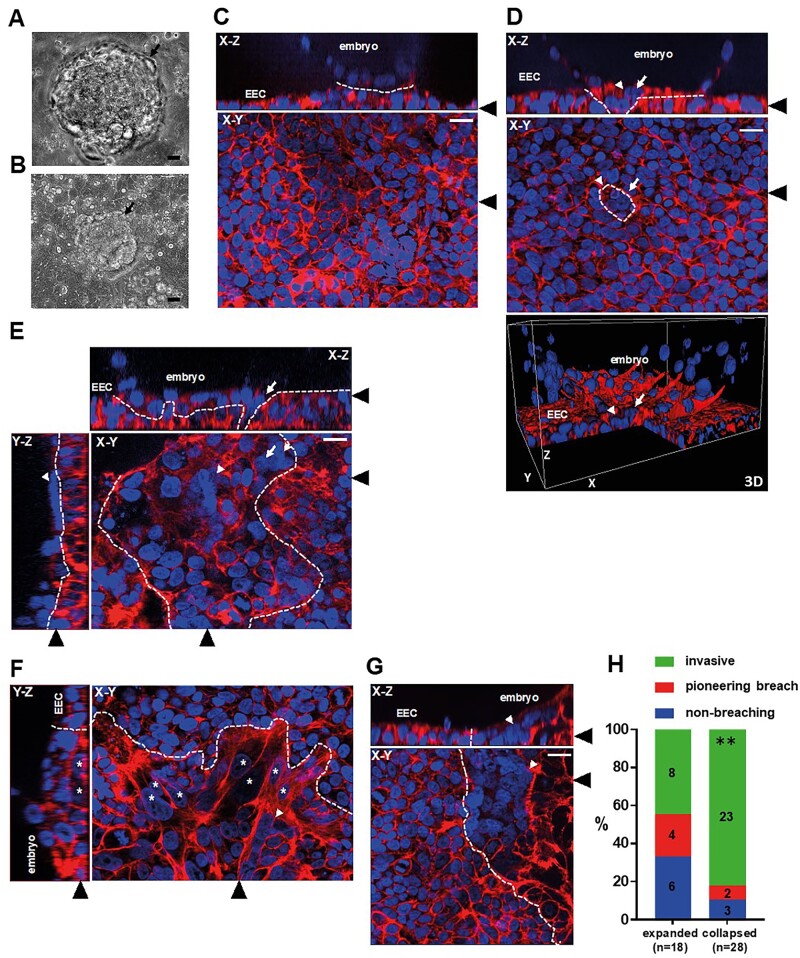
**Syncytiotrophoblast (STB) pioneers embryo invasion of the Ishikawa endometrial epithelial cell (EEC) layer.** (**A, B**) Phase contrast images of embryos attached to Ishikawa EEC layers, showing expanded blastocyst and collapsed blastocyst morphology. Black arrows indicate embryos. Scale bars 20 µm. (**C–G**) Attached embryos were stained with phalloidin (red) and DAPI (blue) to label actin and nuclei, respectively, and imaged by optical sectioning fluorescence microscopy. Panels show X–Y, X–Z and Y–Z planes and a 3D image, as labelled. Ishikawa EEC and embryos are indicated in X–Z and Y–Z planes and the 3D image. Black arrowheads indicate the region of the section for the adjoining panels. White arrows indicate trophoblast breaching of the Ishikawa EEC layer, white arrowheads indicate STB, and white asterisks indicate invasive mononuclear trophoblast. Dotted lines indicate embryo-EEC interface. Scale bars 20 µm. (**H**) Bar graph showing proportions of embryo morphologies (n = 46). **P < 0.01 Chi-squared. DAPI, 4′,6-diamidino-2-phenylindole.

Forty-six blastocysts attached to Ishikawa EEC layers were analysed in this study, all after co-culture for 48 h, from Day 6 post-fertilization to Day 8. Attached embryos exhibited either expanded (18/46, 39%) (Fig. 1A) or collapsed (28/46, 61%) blastocyst morphology (Fig. 1B). We were able to ascertain whether embryos attached in a polar TE- or mural TE-oriented fashion in seven instances, with 5/7 exhibiting polar TE-oriented attachment. Phalloidin labelling of cortical actin filaments combined with DAPI labelling of nuclei enabled single cell resolution of embryos attached to EEC layers. Attached embryos with no clear breaching of the epithelium were observed (Fig. 1C). Embryos in which a defined region of multinuclear STB was mediating initial, pioneering breaching of the epithelium and contacting the underlying substrate were also seen (Fig. 1D and E; Supplementary Movies S1 and S2). In addition, STB was observed in contact with the apical EEC surface (Fig. 1E; Supplementary Movie S2). The majority of embryos had progressed beyond these initial stages to invade laterally into the EEC layer, with morphologically distinct mononuclear and multinuclear outgrowths present (Fig. 1F and G, respectively; Supplementary Movies S3 and S4).

**Figure 2.**
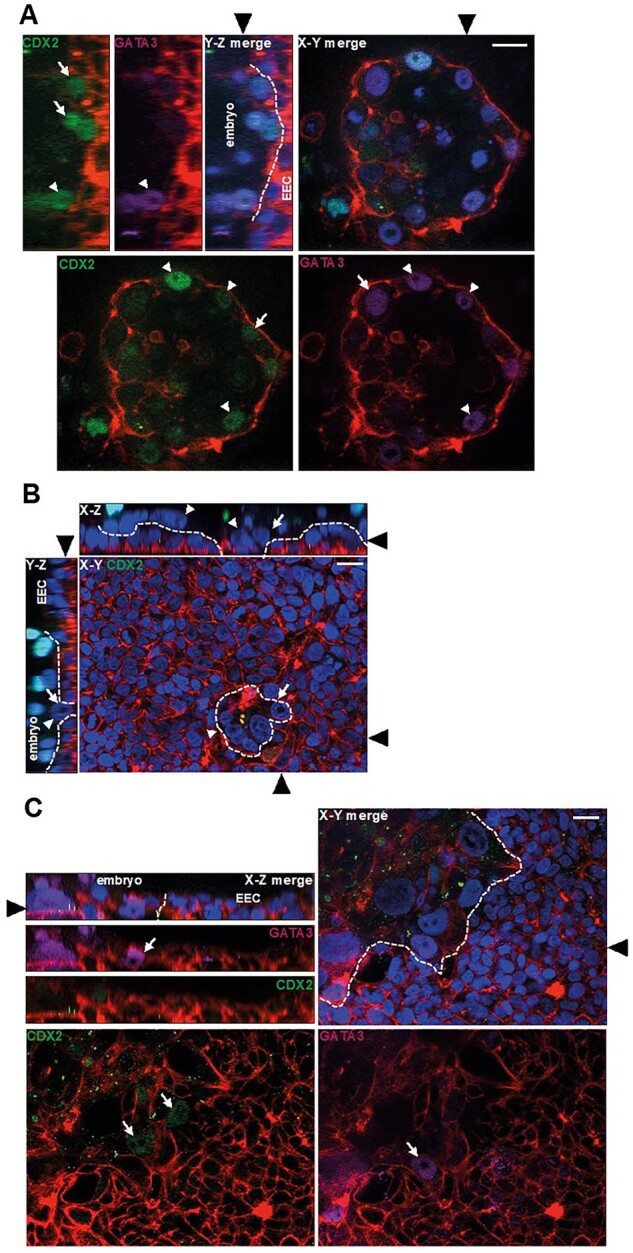
**Trophectoderm (TE) transcription factors at the embryo- endometrial epithelial cell (EEC) interface.** (**A**) Fluorescence optical sections of an attached embryo labelled with phalloidin (red), DAPI (blue), anti-CDX2 (green) and anti-GATA3 antibody (magenta). Y–Z and X–Y planes are shown, and Ishikawa EEC and embryos are indicated Y–Z panel. Black arrowheads indicate the location of the section for the adjoining panels. White arrows show CDX2 or GATA3-positive TE and white arrowheads indicate double-positive CDX2–GATA3-positive TE. Dotted lines indicate embryo-EEC interface. Scale bar 20 µm. (**B**) An attached embryo labelled with phalloidin (red), DAPI (blue) and anti-CDX2 antibody (green) and imaged by optical sectioning fluorescence microscopy. The planes shown in each panel are indicated (X–Z, X–Y) and black arrowheads indicate the location of the section for the adjoining panels. White arrows point to trophoblast breaching of the Ishikawa EEC layer and white arrowheads indicate STB. Dotted lines indicate embryo-EEC interface. Scale bar 20 µm. (**C**) An invasive embryo labelled with phalloidin (red), DAPI (blue), anti-CDX2 (green) and anti-GATA3 antibody (magenta), optical planes indicated on the panels. White arrows indicate CDX2- or GATA3-positive mononuclear trophoblast. Black arrowheads indicate the location of the section for the adjoining panels. Dotted lines indicate embryo-EEC interface. Scale bar 20 µm. DAPI, 4′,6-diamidino-2-phenylindole.

It was observed that 10/18 embryos with expanded blastocyst morphology (56%) were superficially attached to the EEC layer or displayed only pioneering breaching of the epithelium, while 8/18 (44%) had invaded laterally and often extensively into the epithelium. In comparison, 5/28 collapsed embryos (18%) had not breached or exhibited only pioneering breaching, with 23/28 (82%) found to be invasive (Fig. 1H). Collapsed embryos were therefore more likely to have extensively invaded the EEC layer (*P* < 0.01). Importantly, each of the seven pioneering breaching events, observed in six embryos as cells that were in the EEC plane but continuous with an embryonic cell above, consisted of STB protruding through the EEC layer (Figs 1D and E and 2B; Supplementary Movies S1 and S2).

### TE markers in EEC-attached embryos

To characterize the TE/trophoblast cell types in attached embryos, we labelled samples with antibodies to the transcription factors CDX2 and GATA3, which are expressed in this lineage (Hemberger *et al.*, 2020). Co-staining for CDX2 and GATA3 in expanded blastocysts that had not breached the epithelium revealed heterogeneity amongst TE both in contact with, and distal to, EEC; some nuclei were positive only for CDX2, some only for GATA3 and yet others were co-labelled (Fig. 2A). In embryos which had just breached the epithelium, CDX2 was present only in embryonic nuclei above the plane of the EEC layer (Fig. 2B). In collapsed embryos, CDX2 and GATA3 labelling was faintly present in mononuclear trophoblast that had invading extensively into the epithelium (Fig. 2C). Neither transcription factor was detected in STB.

### Multiple STB regions invade into the EEC layer

**Figure 3.**
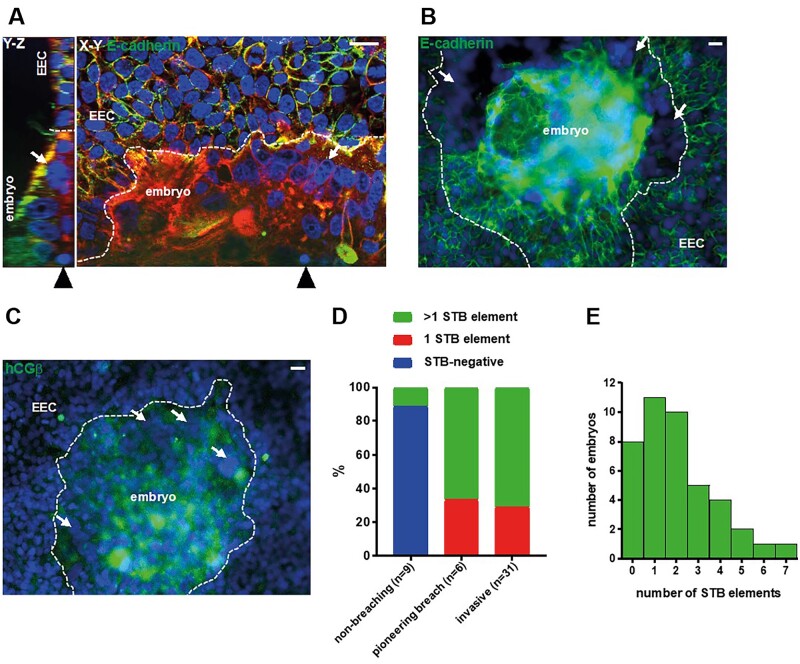
**Multiple regions of syncytiotrophoblast (STB) form at the embryo- endometrial epithelial cell (EEC) interface**. (**A**) Fluorescence optical sections of an invasive embryo labelled with phalloidin (red), DAPI (blue) and anti-E-cadherin (green), panels show Y–Z and X–Y planes. Black arrowheads point to the location of the section for the adjoining panel. White arrows indicate STB. Scale bars 20 µm. (**B**) Fluorescence micrograph of an invasive embryo labelled with DAPI (blue) and E-cadherin (green). White arrows point to distinct regions of STB. Dotted lines indicate embryo-EEC interface. Scale bar 20 µm. (**C**) Fluorescence microscopy image of an invasive embryo labelled with DAPI (blue) and anti-hCGβ (green). White arrows point to distinct regions of STB. Dotted lines indicate embryo-EEC interface. Scale bar 20 µm. (**D**) Bar graph relating STB quantities to embryo invasiveness (n = 46). (**E**) Bar graph of STB quantities in embryos (n = 42; the number of STB elements could not be discerned in four samples). DAPI, 4′,6-diamidino-2-phenylindole.

E-cadherin is present at intercellular junctions in human TE and mononuclear trophoblasts (Alikani, 2005; Aplin *et al.*, 2009), and was used to demarcate mononuclear cells. Multinuclear elements lacking inter-nuclear E-cadherin labelling were observed in contact with EEC at the invading front of embryonic outgrowths (Fig. 3A). This STB contained densely packed nuclei and often occurred at multiple, distinct sites (Fig. 3B). STB and the central area of the embryo were also positive for hCG subunit β (Fig. 3C), the maternal recognition of pregnancy hormone that is secreted by STB from the onset of pregnancy. HLA-G, the canonical marker for invasive mononuclear extravillous trophoblast, was not detected in embryos implanting in this model (Supplementary Fig. S1).

STB, defined as multiple DAPI-stained nuclei within E-cadherin- or phalloidin-stained cell borders, was seen in only 1/9 non-invasive embryos (11.1%). All breaching and invasive embryos contained STB, with approximately two-thirds containing more than one STB element (Fig. 3D). Notably, STB was never observed in TE not in contact with EEC. Analysis of the number of STB elements (multinuclear structures bound by E-cadherin and/or phalloidin staining) present in each embryo demonstrated that up to seven could be found and >70% embryos had one to four distinct STB elements (Fig. 3E).

### Ishikawa EEC layer stimulates STB differentiation in TSC spheroids

**Figure 4.**
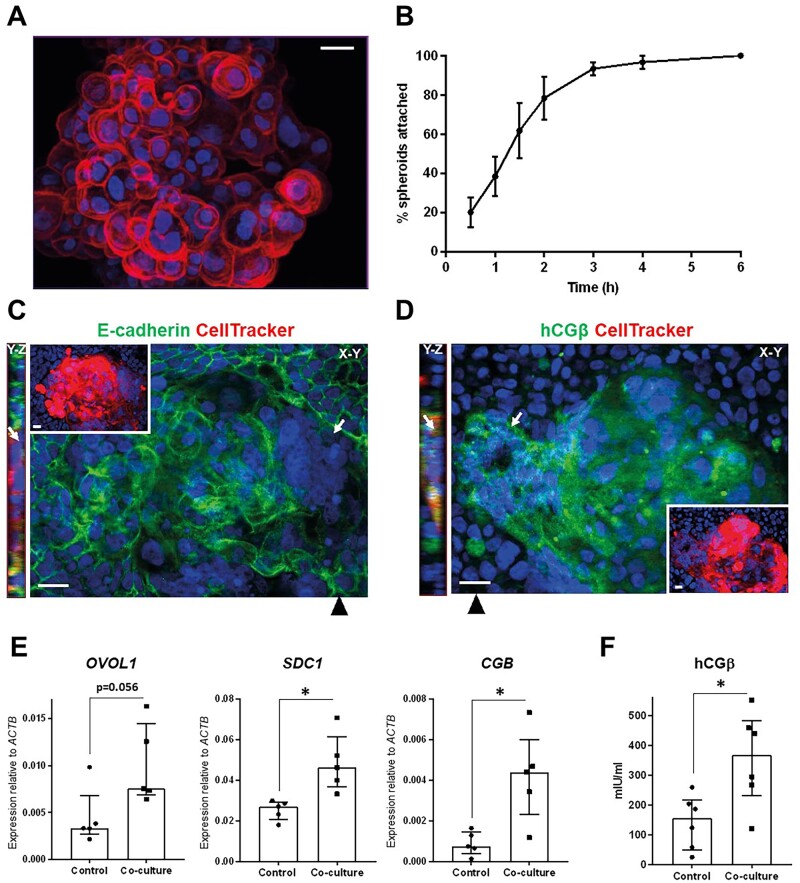
**Syncytiotrophoblast (STB) formation from trophoblast stem cell (TSC) spheroids is promoted by Ishikawa endometrial epithelial cell (EEC) layers.** (**A**) TSC spheroid labelled with phalloidin (red) and DAPI (blue), imaged by optical sectioning fluorescence microscopy and displayed as maximum intensity projection. Scale bar 20 µm. (**B**) TSC spheroid attachment to Ishikawa EEC layers was monitored over 6 h. Three independent repeats were performed (∼20 spheroids per repeat), mean ± SEM plotted on line graph. CellTracker-loaded TSC spheroids attached to Ishikawa EEC layers after 48 h co-culture were labelled with DAPI (blue) and (**C**) anti-E-cadherin (green) or (**D**) anti-hCGβ (green), and optically sectioned by fluorescence microscopy. X–Y and Y–Z planes are shown as indicated, with black arrowheads indicating the location of the Y–Z plane. CellTracker (red) and DAPI labelling is shown in insets. STB is indicated by white arrows. Scale bars 20 µm. (**E**) TSC spheroid-Ishikawa EEC co-cultures and isolated TSC and Ishikawa EEC cultures were lysed after 48 h for real-time quantitative PCR (RT-qPCR) analysis. Expression of STB markers *OVOL1*, *SDC1* and *CGB* was assessed relative to *ACTB* expression. Five experimental repeats, median ± IQR plotted, *P < 0.05 Mann–Whitney. (**F**) Medium was collected from TSC spheroid-Ishikawa EEC co-cultures and isolated TSC and Ishikawa EEC cultures after 48 h for hCGβ ELISA. Six experimental repeats, median ± IQR plotted, * *P* < 0.05 Mann–Whitney. DAPI, 4′,6-diamidino-2-phenylindole.

Human TSC can differentiate to form the major trophoblast subtypes, including STB (Okae *et al.*, 2018), and thus offer a model with greater experimental scope than single embryos for interrogating trophoblast differentiation at implantation. Treatment of TSCs with the previously reported STB differentiation medium induced syncytialization and upregulation of hCGβ at the mRNA and protein level (Supplementary Fig. S2A and B). TSC culture in non-adherent conditions produced blastocyst-sized spheroids of mostly mononuclear cells (Fig. 4A). These were co-cultured in basal medium with Ishikawa EEC layers to model human embryo implantation *in vitro*. TSC spheroids attached to EEC layers within 6 h (Fig. 4B), reflecting the kinetics of blastocyst attachment (Ruane *et al.*, 2020). After 48 h, spheroids had invaded the epithelium with STB at the invasion front (Fig. 4C). Invasive trophoblast was hCGβ-positive, although this was not localized only to invasive multinuclear cells but was seen also in mononuclear trophoblast (Fig. 4D). The expression of STB markers in spheroids attached to EEC layers (co-culture) and those cultured in isolation and attached to tissue culture plastic (control) was compared with determine whether interaction with EEC stimulated STB differentiation. STB genes *CGB* and *SDC1* were significantly upregulated by co-culture with EEC layers (Fig. 4E), while secreted hCGβ levels were also higher in co-cultured TSC spheroids (Fig. 4F). These data suggest that EEC interactions stimulate STB differentiation from multipotent trophoblast.

### Gene networks at the TE-EEC interface

To identify candidate gene networks functioning at the TE-EEC interface, we used single-cell transcriptomic datasets from blastocyst TE (Petropoulos *et al.*, 2016), and bulk transcriptomic data from EEC directly isolated from endometrium (Chi *et al.*, 2020). Cell surface or secreted genes, differentially expressed in receptive (mid-secretory phase) versus non-receptive (proliferative phase) endometrial epithelium (Supplementary Fig. S3A), were used to identify 39 cognate genes in Day 6 TE. The resulting *in silico* TE-EEC interface (Table I), was enriched for extracellular matrix components, peptidases, cell adhesion molecule-binding proteins, signalling receptor-binding proteins and glycosaminoglycan-binding proteins (Supplementary Fig. S3B).

**Table I:** Summary of *in silico* trophectoderm (TE)-endometrial epithelial cell (EEC) interface, hypernetwork analysis and random forest-defined syncytiotrophoblast (STB) classifier status.

EEC gene	Cognate TE gene	TE subset hypernetwork cluster	Classifier of STB differentiation
*LOXL1*	FBLN5*	Polar and mural	Yes
*IGF1*	IGFBP7*	Polar and mural	Yes
*CRISPLD2*	HSP90AB1*	Polar	Yes
*NOG*	NOG*	Polar	Yes
*CP*	*LTF*	Polar	Yes
*TNC*	*NCAN*	Polar	Yes
*NRXN1*	NLGN1*	Mural	Yes
*ALPP*	COL2A1*	Mural	Yes
*SERPINB8*	FURIN*	Mural	Yes
*CST7*	POTEE*	Mural	Yes
*FLNB*	PSEN1*	Mural	Yes
*TGFBR3*	TGFB1*	Mural	Yes
*VCAN*	HAPLN1*	Mural	Yes
*HEXB*	*HEXB*	Mural	Yes
*CSTB*	*CTSH*	Mural	Yes
*SLPI*	*PLSCR1*	Mural	Yes
*AMBP*	*AMBP*	Polar and mural	No
*KLKB1*	*KNG1*	Mural	No
*GUSB*	*CES1*	Mural	No
*THBD*	*F2*	Mural	No
*C4BPA*	*APCS*	Polar and mural	ND^†^
*IFNG*	*IFNG*	Polar and mural	ND^†^
*IFNAR2*	*IFNA2*	Polar and mural	ND^†^
*SERPING1*	*SELE*	Polar and mural	ND^†^
*FGA*	*FGB*	Mural	ND^†^
*SFRP1*	FZD6*	Neither	ND
*DKK3*	UBA52*	Neither	ND
*LAMA1*	FBLN2*	Neither	ND
*DCN*	EGFR*	Neither	ND
*F2*	SERPINE1*	Neither	ND
*CD55*	CD55*	Neither	ND
*PPFIBP2*	*APP*	Neither	ND
*ANXA2*	*CTSB*	Neither	ND
*PROS1*	*F5*	Neither	ND
*ANK3*	*KCNC1*	Neither	ND
*CPE*	*ROBO2*	Neither	ND
*GPLD1*	*APOA4*	Neither	ND
*FGG*	*FGA*	Neither	ND
*APOD*	*APOD*	Neither	ND

* TE gene differentially expressed in TSC-STB differentiation; ND, not determined; ND^†^, not determined as not expressed in TSC/STB.

**Figure 5.**
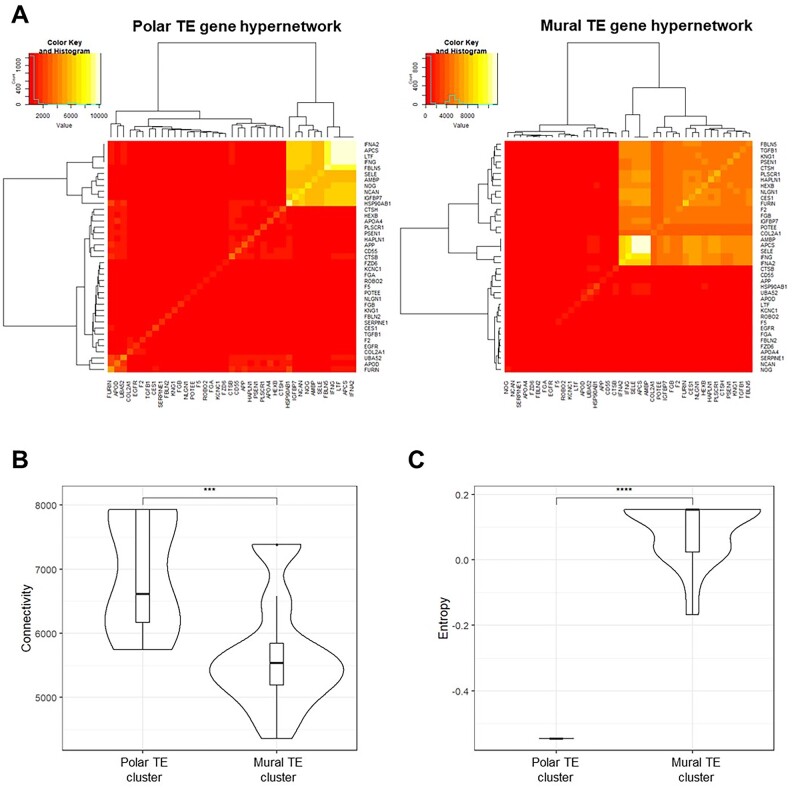
**Polar and mural trophectoderm (TE) gene networks are differentially connected to endometrial epithelial cell (EEC) surface genes in an *in silico* model of the TE-EEC interface.** (**A**) Hypernetwork analysis was performed on EEC-interacting TE genes (n = 39) in polar TE cells (n = 86) and mural TE cells (n = 245), and presented as a heatmap. Eleven genes form the highly connected central cluster in the polar TE hypernetwork and 21 genes form that in the mural TE hypernetwork. (**B**) Connectivity of hypernetwork clusters (the number of pairwise shared correlations) in polar and mural TE is presented as a violin plot. Median line and interquartile range are illustrated in each box, and whiskers illustrate the range with outliers as points. ***P < 0.001 Wilcoxon rank sum test. (**C**) Entropy from the hypernetwork clusters of polar and mural TE (a measure of the organization of gene connections), relative to entropy generated by permuting 1000 hypernetworks of random genes within the respective transcriptomes. Comparison of absolute entropy values between gene networks and subsets therein is not informative connectivity organization. Data are presented in violin plot form as above. ****P < 0.0001 Wilcoxon rank sum test.

Hypernetwork analysis enables clustering of genes based on co-expression within the transcriptome, providing an indication of connectivity in functional gene networks (Pearcy *et al.*, 2016; Gaudelet *et al.*, 2018). Day 6 TE subsets previously defined as polar and mural (Supplementary Fig. S3C) (Petropoulos *et al.*, 2016), enabled us to use hypernetwork analysis to detect differences between polar and mural TE interactions with EEC. A total of 25 distinct genes were identified in the hypernetworks; a cluster of 11 genes from analysis of EEC-interacting polar TE genes, while a 21-gene cluster was found from EEC-interacting mural TE genes (Fig. 5A), with seven genes common between the two clusters (Table I).

The hypernetwork heatmaps suggest enhanced transcriptome connectivity in polar TE compared with mural TE. Quantification of the hypernetwork properties revealed a higher connectivity (number of shared transcriptome correlations) between EEC-interacting polar TE genes compared with those in mural TE (Fig. 5B), and this reflected differences between background transcriptome connectivity in polar and mural TE (Supplementary Fig. S3D). Entropy is a measure of the organization of gene connections within the hypernetwork and is presented relative to background levels. A markedly lower entropy, indicative of a more co-ordinated organization of gene connectivity, was found for EEC-interacting polar TE genes than for those in mural TE (Fig. 5C).

### EEC-coupled TE gene networks are linked to STB differentiation

**Figure 6.**
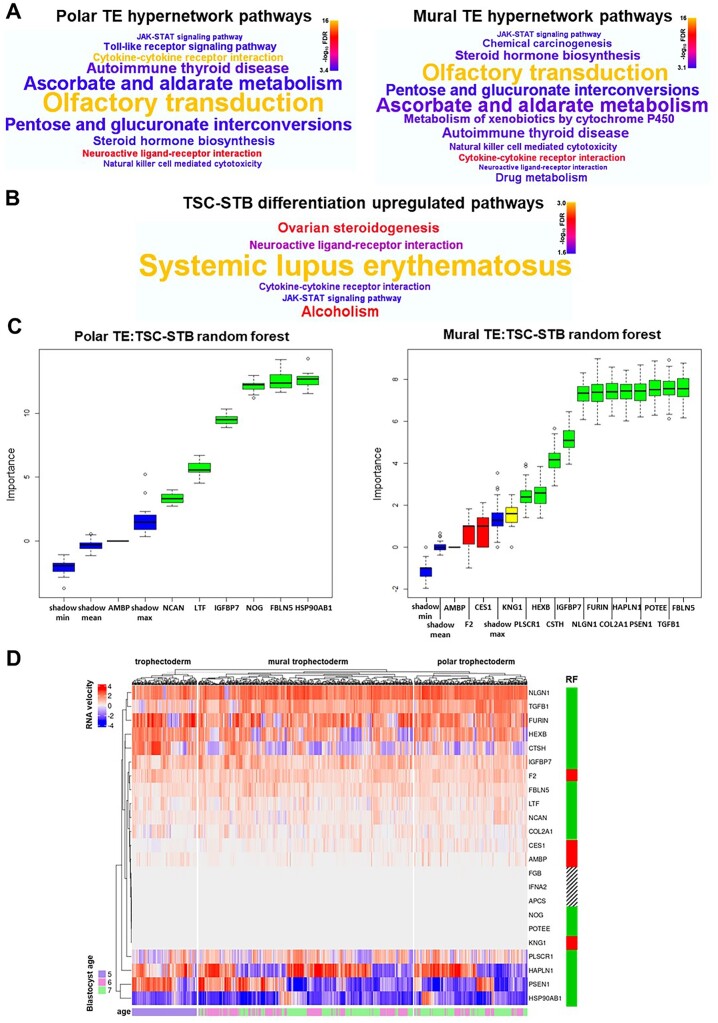
**Endometrial epithelial cell (EEC)-interacting trophectoderm (TE) gene networks are coupled to syncytiotrophoblast (STB) differentiation pathways.** (**A**) Word clouds representing highly enriched (FDR < 0.001) KEGG pathways from over-representation analysis of genes connected within the polar and mural TE hypernetwork clusters, respectively. Word size reflects enrichment ratio, colour relates to significance level. Full list of significantly enriched pathways (FDR < 0.05) in Supplementary Tables SI and SII, respectively. (**B**) Word cloud representing the six most upregulated (FDR < 0.026) KEGG pathways from gene set enrichment analysis of genes differentially expressed upon trophoblast stem cell (TSC) differentiation to STB (*P* < 0.01, TSC n = 4, STB n = 4; Okae *et al.*, 2018). Word size relates to enrichment ratio, colour to significance level. Full list of significantly enriched pathways (FDR < 0.05) in Supplementary Table SIII. (**C**) Boruta plots of random forest machine learning (1000 iterations) showing STB classification importance (TSC n = 4, STB n = 4; Okae *et al.*, 2018) of hypernetwork-clustered EEC-interacting polar and mural TE genes expressed in TSC/STB (n = 7 and n = 16, respectively). Box-whisker plots represent importance *Z*-scores, a metric of informativity of a variable for classification. Green confirms statistical significance in the classification of STB, red indicates a non-informative gene in the classification of STB, yellow indicates a failure to either confirm or reject a gene as informative within the allotted number of random forest runs, and blue demonstrates the background variation of predictive value within the data. (**D**) RNA velocity was measured for each distinct hypernetwork-clustered EEC-interacting TE gene (n = 23, *IFNG* and *SELE* velocity data not available) in each cell of Day 5 TE (polar and mural distinction not available) and Day 6 and 7 polar and mural TE (Petropoulos *et al.*, 2016). These velocity values were clustered within TE cell types in a heatmap, with blastocyst age in days indicated for each TE cell and random forest (RF) classification also indicated for each gene (green, variable of importance in the classification of STB; red, non-informative variable in the classification of STB; barred, importance not determined due to lack of expression in TSC/STB). FDR, false discovery rate.

Pathway analysis of the EEC-coupled polar and mural TE gene hypernetworks (3541 and 2447 genes, respectively) uncovered similar pathways (Fig. 6A; Supplementary Tables SI and SII, respectively). The most enriched pathways consisted of olfactory receptors (olfactory transduction) and cytokines (autoimmune thyroid disease, cytokine–cytokine receptor interaction and natural killer cell-mediated cytotoxicity). Glucuronosyltransferases were also highly prevalent in enriched pathways including steroid hormone biosynthesis, within which central progesterone metabolism genes *HSD3B2*, *CYP17A1*, *CYP11B2* and *AKR1C1* were present in the hypernetworks. Steroid hormone biosynthesis, especially progesterone synthesis, is a key pathway for endocrine activity in STB (Okae *et al.*, 2018). Remarkably, comparing EEC-coupled TE pathways to a gene set enrichment analysis of TSC differentiation to STB (9728 genes; Okae *et al.*, 2018) demonstrated that 4/6 most highly upregulated pathways in STB differentiation (false discovery rate (FDR < 0.026)) were highly significantly enriched in both polar and mural hypernetworks (FDR < 0.001): steroid hormone biosynthesis/ovarian steroidogenesis (overlapping pathways), cytokine–cytokine receptor interaction, neuroactive ligand-receptor interaction and the JAK-STAT signalling pathway (Fig. 6B; Supplementary Table SIII).

To test whether the TE genes in our *in silico* interface were linked to STB differentiation, we applied RF machine learning. This quantitative approach revealed that 80% (16/20) of the distinct EEC-interacting genes in polar and mural TE could be considered variables of importance in classifying STB (Fig. 6C and Table I), with *FBLN5* and *IGFBP7* appearing as significant in both polar and mural TE. RNA velocity was assessed for TE genes in the *in silico* model as a direct measure of the rate of change of expression (La Manno *et al.*, 2018). The genes classified as important variables by RF exhibited dynamic gene expression in TE cells from Day 5 to 7, clustering at the upper and lower regions of the heatmap compared with genes that were not classified as variables of importance that generally clustered in the middle with little dynamic expression (Fig. 6D), therefore, further implicating functionality for these genes in TE development. Hypernetworks, pathway analysis, RF machine learning and RNA velocity analysis in our *in silico* model together suggest that EEC-interacting genes in both polar and mural TE are linked to functional gene networks involved in STB differentiation.

## Discussion

Embryo implantation into the uterine endometrium is essential for human development, and this process begins with blastocyst attachment to the endometrial epithelium and breaching of this barrier (Aplin and Ruane, 2017). Here, *in vitro* and *in silico* models of the epithelial phase of human embryo implantation reveal for the first time that interactions with EEC promote TE differentiation to STB, which then goes on to breach the endometrial epithelium. These findings establish the importance of EEC interactions to the development of trophoblast lineages at implantation. To further investigate the pattern of trophoblast development at implantation, sequential interactions with endometrial epithelium, subjacent stromal matrix and decidual cell types must be considered. Understanding these processes will illuminate infertility and obstetric disease mechanisms thought to be underpinned by defective trophoblast development at implantation (Aplin *et al.*, 2020).

Blastocysts that had attached to Ishikawa EEC layers after 48 h co-culture, from Day 6 to 8, revealed a range of implantation stages, from those attached to the EEC apical surface to those that had extensively invaded into the epithelium. Blastocyst cavity collapse is consistently observed by Day 8 in embryos cultured in isolation (Deglincerti *et al.*, 2016; Shahbazi *et al.*, 2016), and consistent with this, most embryos here displayed collapsed cavities and invasive morphology. Those embryos that remained expanded and did not breach the epithelium may reflect slower implantation kinetics or implantation failure. STB was observed in all invasive embryos and consistently pioneered breaching of the epithelium, with these breaching events appearing to form between cells of the EEC layer. STB may negotiate invasion between EEC via the formation of junctional complexes between trophoblast and lateral EEC membranes, which have been seen in ultrastructural studies in both human and monkey (Enders *et al.*, 1983; Smith *et al.*, 1987; Bentin-Ley *et al.*, 2000). Both mononuclear trophoblast and STB were present at the leading edge of embryos invading laterally into the EEC layer, suggesting that the initial stromal phase of invasion may not be driven solely by STB (James *et al.*, 2012; Knofler *et al.*, 2019; Turco and Moffett, 2019). However, the 2D nature of the *in vitro* EEC model and the absence of underlying matrix and stromal cell types may have influenced invasive cell behaviour. The well-differentiated endometrial adenocarcinoma-derived Ishikawa cell line provides a consistent, polarized, receptive 2D epithelial model that is difficult to generate using primary EEC (Campbell *et al.*, 2000; Singh and Aplin, 2015; Ruane *et al.*, 2017), nevertheless 3D cultures of primary endometrial cells are required to better recapitulate implantation. Moreover, using high grade blastocysts may also improve *in vitro* models of implantation.

Our observational evidence from embryo-EEC cultures and experimental evidence from TSC spheroid-EEC cultures suggest that interactions with the apical surface of EEC promotes extensive STB formation before this cell type invades between cells of the EEC layer. Formation of STB has been seen in Day 8 embryos cultured in the absence of endometrial cells (Deglincerti *et al.*, 2016; Shahbazi *et al.*, 2016), while syncytialization is well established to occur spontaneously in primary placental trophoblast (Kliman et al., 1986). Our *in silico* models provide independent evidence that EEC interactions stimulate differentiation pathways in TE, and this may act to promote STB formation and ensure timely breaching of the epithelium. The STB we observed in both implanting embryos and the TSC spheroid implantation model was invasive, and this primary STB present at implantation contrasts with the canonical STB that forms from villous cytotrophoblasts and bounds the villi at later stages of placentation (Aplin, 2010; James *et al.*, 2012; Yabe *et al.*, 2016). TSC spheroid interactions with Ishikawa cell layers produced invasive STB that was morphologically very similar to that formed from embryos, thus leading us to favour a model in which the primary STB phenotype is induced by interactions with EEC. Conversely, formation directly from TE as opposed to from cytotrophoblast could also contribute to the unique phenotype of primary STB. EEC-induced invasive trophoblast differentiation from TE was previously shown in mouse blastocysts (Ruane *et al.*, 2017), implicating evolutionary conservation of this mechanism at the onset of interstitial implantation.

We modelled the TE-EEC interface using network biology techniques as a discovery-phase approach to identify candidate functional gene networks that could be assessed in future *in vitro* studies. A prior network biology approach used whole embryo and whole endometrium microarray transcriptomes to identify key gene networks, including adhesion, extracellular matrix and cytokine genes (Altmae *et al.*, 2012). While we found some of the same genes (*APOD*, *VCAN*, *FBLN2*, *LAMA1*, *SERPINE1*, *TGFB1*), our focus on EEC interactions with polar and mural TE subpopulations using RNAseq data provided a model with enhanced resolution. Furthermore, hypernetwork analysis enabled enrichment of functional gene networks, which we have previously used to investigate ICM and TE lineage allocation from the eight-cell blastomere stage in human embryos (Smith *et al.*, 2019). The expression correlation matrices underlying hypernetworks capture gene networks that relate to functional units within the transcriptome, with the sensitivity of this correlation approach lending itself to highly dynamic biological systems (Pearcy *et al.*, 2016; Gaudelet *et al.*, 2018; Chantzichristos *et al.*, 2021). Increased resolution of *in silico* implantation models could be obtained by stratifying EEC populations, as have been recently characterized using spatial transcriptomics (Garcia-Alonso *et al.*, 2021). Indeed, comparison with that dataset revealed that all but one (*NRXN1*) of the EEC genes identified as candidates to promote TE differentiation to STB were expressed in the luminal EEC populations to which blastocysts attach *in vivo*.

Accordingly, pathway analysis of the hypernetworks linked to EEC-interacting TE genes uncovered a striking resemblance to the pathways activated upon STB differentiation from TSC (Okae *et al.*, 2018), a process that we directly observed when TSC spheroids implanted into Ishikawa EEC layers. Hormone production, including progesterone, is a classical function of STB (Costa, 2016), so the coupling of EEC-interacting TE genes to steroid hormone biosynthetic pathways is strongly suggestive of STB differentiation. The presence of cytokines, predominantly chemokines and interleukins, in the EEC-coupled hypernetworks may also be a marker of STB, since immunomodulation is another key STB attribute (Du *et al.*, 2014). The common signalling pathways seen in TSC-STB differentiation and the TE hypernetworks consisted of JAK-STAT and G-protein-coupled receptors of the neuroactive ligand-receptor interaction pathway, and both of these have been implicated in transducing STB differentiation signals (Knerr *et al.*, 2005; Leduc *et al.*, 2012). RF machine learning was used as a quantitative measure to test the hypothesis that the functionally enriched TE genes at the interface with EEC were linked to STB differentiation, and this analysis yielded a strong signal favouring the hypothesis. Furthermore, dynamic expression indicative of function was demonstrated for many of these genes by RNA velocity analysis (La Manno *et al.*, 2018). Delineating the exact pathways signalling from the interface to differentiation processes was not possible with these approaches, however, the regulation of integrin and insulin-like growth factor (IGF) signalling is hinted at through the robust identification of integrin ligand *FBLN5* and IGF-binding protein *IGFBP7* as common functional factors in both polar and mural TE. Prior *in vitro* studies have shown these pathways to function during blastocyst-EEC attachment at implantation (Kang *et al.*, 2014; Green *et al.*, 2015), indeed IGF can signal through JAK-STAT (Zhou et al., 2021) and there is evidence of co-regulation between IGF and integrin signalling (Takada *et al.*, 2017).

Single cell transcriptomic discrimination of TE subpopulations have linked polar TE with upregulation of STB differentiation genes (Petropoulos *et al.*, 2016; Lv *et al.*, 2019). Here, we used these TE subpopulations and found increased levels and organization of connectivity in EEC-interacting polar TE gene networks. Gene network properties of connectivity and organization are indicative of cell differentiation state; high network organization (measured as low entropy) characterizes reduced regulatory cross-talk and pathway redundancy in more differentiated cells (Banerji *et al.*, 2013; Teschendorff and Enver, 2017). Our analysis, therefore, corroborates polar TE as more differentiated than mural TE and suggests that interactions with EEC may be more strongly linked to cell differentiation networks in polar TE than in mural TE. Polar TE-oriented attachment to endometrial epithelium was observed here and in previous studies (Bentin-Ley *et al.*, 2000; Aberkane *et al.*, 2018), and embryonic pole-oriented stromal invasion was observed *in vivo* (Hertig *et al.*, 1956). The more differentiated state of EEC-attached polar TE could therefore lead to polar-oriented STB invasion. In contrast, our pathway, RF and RNA velocity analyses found overall similarities between polar and mural TE. Moreover, in embryos we observed multiple independent STB regions in contact with Ishikawa cells. Together, our findings lead to a model whereby STB forms from EEC-attached polar and polar-proximal mural TE before initiating invasion into the endometrium. The corollary of this model could be that distal mural TE not engaged with EEC gives rise to TSCs. This scenario is opposite to that in mice, where mural TE initiates invasion and polar TE gives rise to multipotent trophoblast that go on to form the placenta (Sutherland, 2003). Studies of differential phagocytosis activity between polar and mural TE support this inversion of embryonic pole functionality between mouse and human blastocysts (Rassoulzadegan *et al.*, 2000; Li *et al.*, 2016).

Further work with sophisticated *in vitro* models consisting of multiple maternal cell types are required to pursue some of the outcomes of this study and to fully characterize the establishment of trophoblast populations at implantation. Failure of this process is likely behind many of the recurrent implantation failures seen in assisted reproduction patients and may also contribute to cases of recurrent miscarriage (Koot *et al.*, 2011). Furthermore, there is increasing awareness that later stage obstetric pathologies have their origin in implantation and early pregnancy (Rabaglino *et al.*, 2015; Garrido-Gomez *et al.*, 2017; Than *et al.*, 2018; Aplin *et al.*, 2020), and understanding endometrial shaping of trophoblast development is central to developing new treatments.

## Data availability

Much of the data underlying this article are available within the article and in its online supplementary material. The totality of the data underlying this article will be shared on reasonable request to the corresponding author.

## Supplementary Material

deac008_Supplementary_Figure_S1

deac008_Supplementary_Figure_S2

deac008_Supplementary_Figure_S3

deac008_Supplementary_Table_S1

deac008_Supplementary_Table_S2

deac008_Supplementary_Table_S3

deac008_Supplementary_Movie_S1

deac008_Supplementary_Movie_S2

deac008_Supplementary_Movie_S3

deac008_Supplementary_Movie_S4
