# Supplementary material for: Trophectoderm differentiation to invasive syncytiotrophoblast is promoted by endometrial epithelial cells during human embryo implantation
Source: Hum Reprod. 2022 Jan 26;37(4):777–92. doi: 10.1093/humrep/deac008 (PMC9398450; doi:10.1093/humrep/deac008)
Supplement: deac008_Supplementary_Figure_S1 [file deac008_supplementary_figure_s1.pdf]

Human Reproduction, pp. 1–1, 2022
https://doi.org/10.1093/humrep/deac008

human reproduction

SUPPLEMENTARY DATA

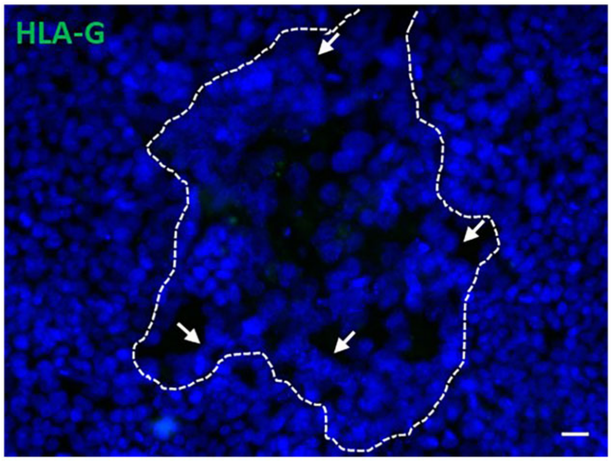

Supplementary Figure S1. HLA-G is not expressed in embryos attached to Ishikawa endometrial epithelial cell (EEC) layers. Fluorescence micrograph of an invasive embryo labelled with DAPI (blue) and HLA-G (green). White arrows point to regions of syncytiotrophoblast. Dotted lines indicate embryo-EEC interface. Scale bar 20 μm. DAPI, 4′,6-diamidino-2-phenylindole.
