# Supplementary material for: Trophectoderm differentiation to invasive syncytiotrophoblast is promoted by endometrial epithelial cells during human embryo implantation
Source: Hum Reprod. 2022 Jan 26;37(4):777–92. doi: 10.1093/humrep/deac008 (PMC9398450; doi:10.1093/humrep/deac008)
Supplement: deac008_Supplementary_Figure_S2 [file deac008_supplementary_figure_s2.pdf]

Human Reproduction, pp. 1–1, 2022
https://doi.org/10.1093/humrep/deac008

human reproduction

SUPPLEMENTARY DATA

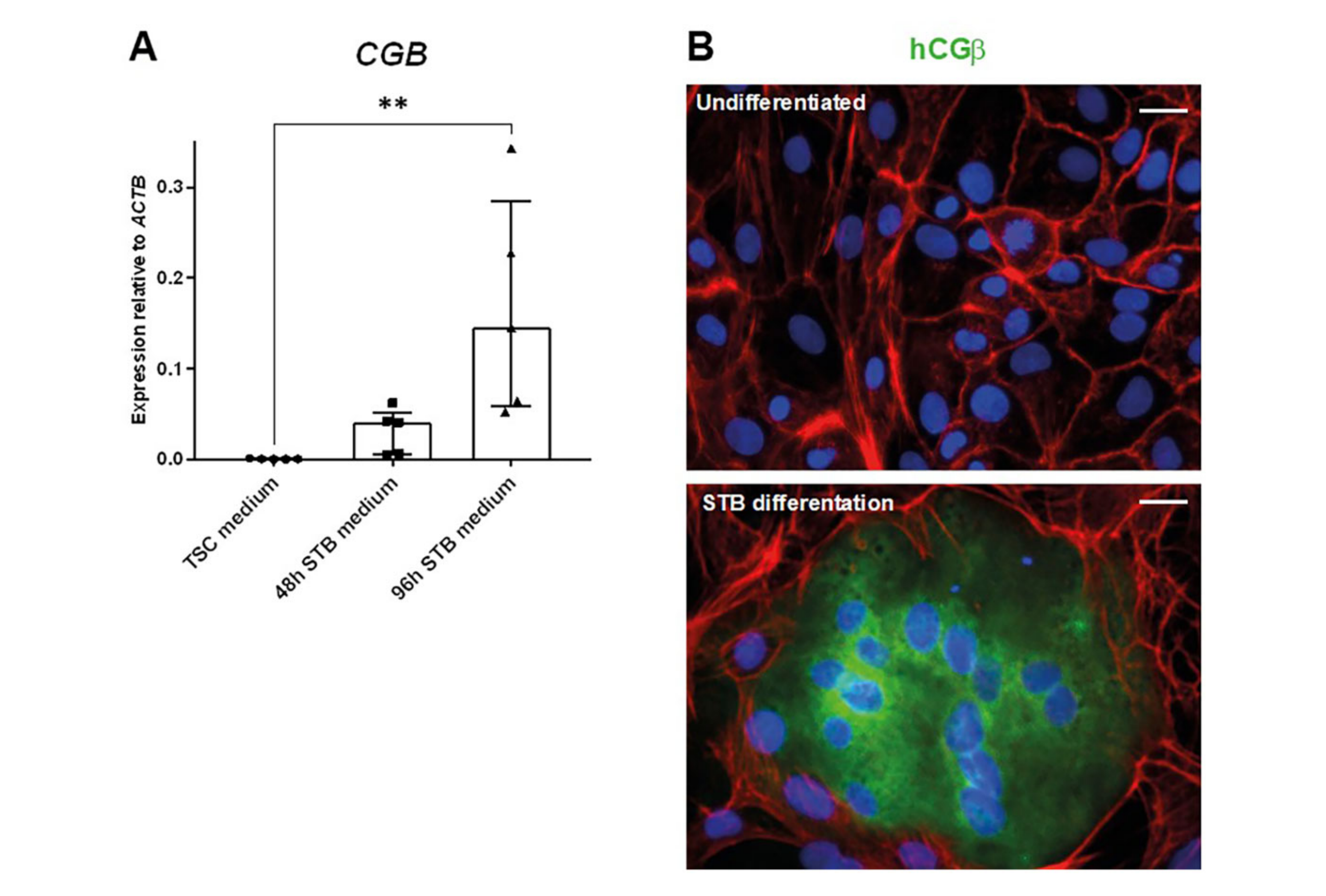

Supplementary Figure S2. Trophoblast stem cell (TSC) can be induced to differentiate to syncytiotrophoblast (STB). (**A**) TSC were cultured in STB differentiation medium for 48 and 96 h, and were compared with TSC cultured in TSC medium. Cells were lysed for real-time quantitative PCR (RT-qPCR) and expression of STB marker *CGB* was expressed relative to *ACTB*. Five experimental repeats, median ± IQR plotted, $^{**}P < 0.01$ Mann–Whitney. (**B**) TSC were cultured in TSC medium and in STB differentiation medium for 96 h. Cells were labelled with phalloidin (red), DAPI (blue), and anti-hCGβ (green). Scale bars 20 µm. DAPI, 4′,6-diamidino-2-phenylindole.
