# Supplementary material for: Trophectoderm differentiation to invasive syncytiotrophoblast is promoted by endometrial epithelial cells during human embryo implantation
Source: Hum Reprod. 2022 Jan 26;37(4):777–92. doi: 10.1093/humrep/deac008 (PMC9398450; doi:10.1093/humrep/deac008)
Supplement: deac008_Supplementary_Figure_S3 [file deac008_supplementary_figure_s3.pdf]

Human Reproduction, pp. 1–1, 2022
https://doi.org/10.1093/humrep/deac008

human reproduction

SUPPLEMENTARY DATA

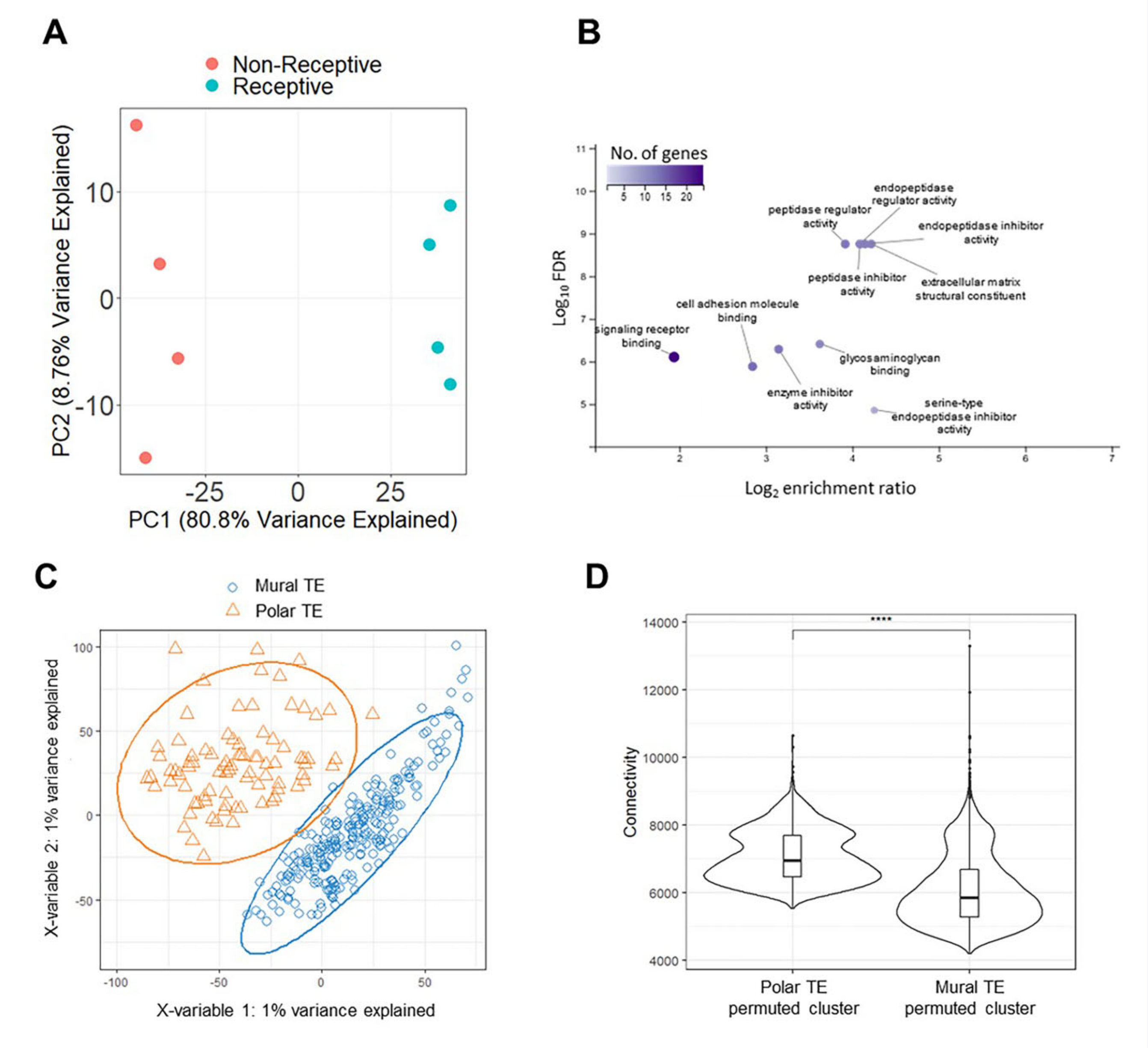

Supplementary Figure S3. Characteristics of the *in silico* trophectoderm (TE)-endometrial epithelial cell (EEC) interface. (**A**) Principal component analysis (PCA) of primary EEC transcriptomes from eight patients, four proliferative phase (receptive) samples (red) and four mid-secretory-phase (non-receptive) samples (blue) (Chi *et al.*, 2020). PCA calculated on differentially regulated genes (DEGs) (n = 2131, $P < 0.01$ to 8.53E−8) between proliferative and mid-secretory samples. Genes that were downregulated in mid-secretory EEC were not omitted in order to prevent introducing directional bias to the gene networks identified downstream. (***B***) Molecular function gene ontologies for TE-EEC interface genes, presented as false discovery rate (FDR) relative to gene-molecular function enrichment ratio. (**C**) Partial least squares-discriminant analysis (PLS-DA) of polar and mural TE (Petropoulos *et al.*, 2016) performed on 331 whole TE transcriptomes (n = 142 780), demonstrating separation of TE into polar and mural based on a small fraction of the transcriptomes (Petropoulos *et al.*, 2016; Lv *et al.*, 2019). (**D**) Violin plots of background levels of polar and mural TE gene connectivity, as measured by permuting 1000 hypernetworks of random genes within the transcriptomes. Median line and interquartile range are illustrated in each box while whiskers illustrate the range, with outliers as points. ***$P < 0.001$ Wilcoxon rank sum test.
