# Supplementary material for: Trophectoderm differentiation to invasive syncytiotrophoblast is promoted by endometrial epithelial cells during human embryo implantation
Source: Hum Reprod. 2022 Jan 26;37(4):777–92. doi: 10.1093/humrep/deac008 (PMC9398450; doi:10.1093/humrep/deac008)
Supplement: deac008_Supplementary_Table_S1 [file deac008_supplementary_table_s1.pdf]

Human Reproduction, pp. 1–1, 2022
https://doi.org/10.1093/humrep/deac008

human reproduction

SUPPLEMENTARY DATA

Supplementary Table SI **Enriched KEGG pathways (FDR < 0.05) from over-representation analysis of 3541 genes in the hypernetwork connected to EEC-interacting polar TE genes.**

Gene set	Description	Size	Ratio	FDR
hsa04740	Olfactory transduction	448	5.753	<1.00E−16
hsa04060	Cytokine–cytokine receptor interaction	294	2.700	<1.00E−16
hsa04080	Neuroactive ligand-receptor interaction	277	2.476	2.00E−11
hsa05320	Autoimmune thyroid disease	53	3.513	3.97E−05
hsa04650	Natural killer cell-mediated cytotoxicity	131	2.394	1.17E−04
hsa00040	Pentose and glucuronate interconversions	34	4.035	1.27E−04
hsa04620	Toll-like receptor signalling pathway	104	2.544	1.56E−04
hsa00140	Steroid hormone biosynthesis	60	3.103	1.71E−04
hsa00053	Ascorbate and aldarate metabolism	27	4.355	1.73E−04
hsa04630	JAK-STAT signalling pathway	162	2.117	3.85E−04
hsa04622	RIG-I-like receptor signalling pathway	70	2.660	1.44E−03
hsa04623	Cytosolic DNA-sensing pathway	63	2.644	3.53E−03
hsa04742	Taste transduction	83	2.361	4.74E−03
hsa04657	IL-17 signalling pathway	93	2.213	8.06E−03
hsa00860	Porphyrin and chlorophyll metabolism	42	2.800	1.56E−02
hsa05150	*Staphylococcus aureus* infection	56	2.450	2.35E−02
hsa04062	Chemokine signalling pathway	189	1.711	2.62E−02
hsa00830	Retinol metabolism	67	2.194	4.65E−02

FDR, false discovery rate; EEC, endometrial epithelial cell; TE, trophectoderm.
