# Supplementary material for: Trophectoderm differentiation to invasive syncytiotrophoblast is promoted by endometrial epithelial cells during human embryo implantation
Source: Hum Reprod. 2022 Jan 26;37(4):777–92. doi: 10.1093/humrep/deac008 (PMC9398450; doi:10.1093/humrep/deac008)
Supplement: deac008_Supplementary_Table_S2 [file deac008_supplementary_table_s2.pdf]

Human Reproduction, pp. 1–1, 2022
https://doi.org/10.1093/humrep/deac008

human reproduction

SUPPLEMENTARY DATA

Supplementary Table SII **Enriched Kyoto Encyclopedia of Genes and Genomes (KEGG) pathways (false discovery rate (FDR) < 0.05) from over-representation analysis of 2447 genes in the hypernetwork connected to endometrial epithelium-interacting mural trophectoderm genes.**

Gene set	Description	Size	Ratio	FDR
hsa04740	Olfactory transduction	448	6.861	<1.00E−16
hsa04060	Cytokine–cytokine receptor interaction	294	2.815	8.31E−11
hsa00980	Metabolism of xenobiotics by cytochrome P450	76	3.695	4.70E−05
hsa00053	Ascorbate and aldarate metabolism	27	6.021	4.79E−05
hsa05320	Autoimmune thyroid disease	53	4.183	7.61E−05
hsa00140	Steroid hormone biosynthesis	60	3.941	7.61E−05
hsa04650	Natural killer cell-mediated cytotoxicity	131	2.820	8.07E−05
hsa00982	Drug metabolism	72	3.489	1.68E−04
hsa05204	Chemical carcinogenesis	82	3.244	2.36E−04
hsa00040	Pentose and glucuronate interconversions	34	4.781	2.47E−04
hsa04080	Neuroactive ligand-receptor interaction	277	2.081	2.47E−04
hsa04630	JAK-STAT signalling pathway	162	2.372	7.52E−04
hsa00830	Retinol metabolism	67	3.309	7.52E−04
hsa04622	RIG-I-like receptor signalling pathway	70	3.167	1.21E−03
hsa04623	Cytosolic DNA-sensing pathway	63	3.284	1.31E−03
hsa00860	Porphyrin and chlorophyll metabolism	42	3.871	1.55E−03
hsa00983	Drug metabolism	79	2.432	4.35E−02
