# Supplementary material for: Trophectoderm differentiation to invasive syncytiotrophoblast is promoted by endometrial epithelial cells during human embryo implantation
Source: Hum Reprod. 2022 Jan 26;37(4):777–92. doi: 10.1093/humrep/deac008 (PMC9398450; doi:10.1093/humrep/deac008)
Supplement: deac008_Supplementary_Table_S3 [file deac008_supplementary_table_s3.pdf]

Human Reproduction, pp. 1–1, 2022
https://doi.org/10.1093/humrep/deac008

human reproduction

SUPPLEMENTARY DATA

Supplementary Table SIII **Enriched Kyoto Encyclopedia of Genes and Genomes (KEGG) pathways (false discovery rate (FDR) < 0.05) from gene set enrichment analysis (positive normalized enrichment score (NES), pathway upregulated; negative NES, pathway downregulated) of 9728 genes differentially expressed upon trophoblast stem cell (TSC) differentiation to syncytiotrophoblast (STB) (*P* < 0.01, TSC n = 4, STB n = 4; Okae *et al.*, 2018).**

Gene set	Description	Size	NES	FDR
hsa05322	Systemic lupus erythematosus	47	3.031	<1.00E−04
hsa05034	Alcoholism	87	2.150	3.10E−03
hsa04913	Ovarian steroidogenesis	21	2.080	3.62E−03
hsa04080	Neuroactive ligand-receptor interaction	56	1.941	1.28E−02
hsa04060	Cytokine–cytokine receptor interaction	79	1.841	1.99E−02
hsa04630	JAK-STAT signalling pathway	77	1.785	2.51E−02
hsa04610	Complement and coagulation cascades	28	1.733	2.99E−02
hsa04978	Mineral absorption	23	1.720	2.89E−02
hsa05222	Small cell lung cancer	69	−1.677	4.89E−02
hsa05219	Bladder cancer	31	−1.691	4.57E−02
hsa03460	Fanconi anaemia pathway	36	−1.693	4.97E−02
hsa05217	Basal cell carcinoma	39	−1.719	3.93E−02
hsa04310	Wnt signalling pathway	90	−1.737	3.68E−02
hsa04974	Protein digestion and absorption	32	−1.748	3.68E−02
hsa04390	Hippo signalling pathway	98	−1.754	4.08E−02
hsa03030	DNA replication	35	−1.793	2.93E−02
hsa04218	Cellular senescence	114	−1.808	3.08E−02
hsa03440	Homologous recombination	31	−1.854	2.08E−02
hsa04110	Cell cycle	110	−2.115	<1.00E−04
